# Electronic Structure Calculations on Endohedral Complexes of Fullerenes: Reminiscences and Prospects

**DOI:** 10.3390/molecules28031384

**Published:** 2023-02-01

**Authors:** Jerzy Cioslowski

**Affiliations:** Institute of Physics, University of Szczecin, Wielkopolska 15, 70-451 Szczecin, Poland; jerzy.cioslowski@usz.edu.pl

**Keywords:** fullerenes, endohedral complexes, electronic structure calculations, host–guest interactions

## Abstract

The history of electronic structure calculations on the endohedral complexes of fullerenes is reviewed. First, the long road to the isolation of new allotropes of carbon that commenced with the seminal organic syntheses involving simple inorganic substrates is discussed. Next, the focus is switched to author’s involvement with fullerene research that has led to the in silico discovery of endohedral complexes. The predictions of these pioneering theoretical studies are juxtaposed against the data afforded by subsequent experimental developments. The successes and failures of the old and modern quantum-chemical calculations on endohedral complexes are summarized and their remaining deficiencies requiring further attention are identified.

## 1. Introduction

Contrary to common belief, neither urea was the first organic compound ever synthesized from inorganic substrates nor benzene was the first aromatic compound ever isolated. In 1825, Leopold Gmelin extracted croconic acid C${}_{5}$H${}_{2}$O${}_{5}$ and potassium croconate dihydrate K${}_{2}$C${}_{5}$O${}_{5}\cdot$2H${}_{2}$O from the reaction mixture obtained by heating potassium carbonate with carbon [1]. On account of the intense yellow and orange colors exhibited by these compounds and their derivatives, he derived their names from the Ancient Greek word κρόκος that means either saffron or egg yolk. Nine years later, Justus Freiherr von Liebig studied the reaction of metallic potassium with carbon monoxide [2]. He observed the metal first becoming silver-colored, then gray, and finally deep black. When the absorption of carbon monoxide was complete, a vigorous reaction suddenly turned the black material into a stable product with the appearance of a gray solid. Subsequent investigations have proven this substance to be the hexapotassium salt of hexahydroxybenzene with the composition K${}_{6}$C${}_{6}$O${}_{6}$ [3].

These two seminal syntheses have heralded the commencement of the golden era of organic chemistry that extended through the remaining part of the nineteenth century. It was characterized by a rapid succession of discoveries of new functional groups, new classes of compounds, and new reactions that interrelated them. The synthesis of urea (1828) by Friedrich Wöhler [4], the isolation of “bicarburet of hydrogen” (i.e., benzene) from the oily residue encountered in the production of illuminating gas (1825) by Michael Faraday [5], the discovery of the asymmetric carbon (1874) by Jacobus Henricus “Henry” van ’t Hoff Jr. [6] and Joseph Achille Le Bel [7], and the formulation of the Spannung (strain) theory (1885) by Johann Friedrich Wilhelm Adolf von Baeyer [8] are just a few examples of the abundant milestones of that era.

With organic chemistry inevitably becoming a mature science, the pace of such fundamental discoveries slowed down considerably in the twentieth century. Nevertheless, a great deal of exciting developments took place, including syntheses of many compounds with unusual geometries and arrangements of bonds. Among them, 1,3,5,7-cyclooctatetraene (the first non-aromatic higher annulene obtained in 1911 by Richard Martin Willstätter) [9], atypical polycyclic aromatic hydrocarbons (such as kekulene also known as “superbenzene” [10] and helicenes [11]), bicyclo[1.1.0]butane and [1.1.1]propellane (the highly strained hydrocarbons synthesized by Kenneth Berle Wiberg and his collaborators) [12,13], certain cage hydrocarbons (such as prismane also known as “Ladenburg benzene” [14] and twistane [15]), the diamondoids (e.g., adamantane [16], iceane also known as wurtzitane [17,18], and diamantane also known as congressane [19]), and the Platonic hydrocarbons (i.e., cubane [20] and dodecahedrane [21]), are worth mentioning. Even more importantly, entirely new classes of compounds, such as those comprising interlocked subunits (e.g., catenanes [22,23]) and those involving host–guest molecular assemblies formed upon entrapment of cations and anions by crown ethers [24] and cryptands [25], have emerged thanks to synthetic efforts of multiple research groups.

These remarkable achievements of organic and supramolecular chemists have created the impression of little room being left for further discoveries of radically new types of substances composed predominantly of carbon. In a scenario reminiscent of the common belief of the pre-quantum era that most of the grand underlying principles of physics had been already firmly established, this impression set a stage for one of the greatest surprises in chemistry of the twentieth century. It occurred in 1990 when, validating the earlier conjecture based solely on mass-spectroscopic evidence [26], Wolfgang Krätschmer, Lowell D. Lamb, Konstantinos Fostiropoulos, and Donald R. Huffman isolated the C${}_{60}$ fullerene and its higher congeners from carbon soot produced by the evaporation of graphite electrodes in low-pressure helium [27]. Their discovery had both symbolic meaning and significant practical consequences. On one hand, it embodied the unification of the aforementioned archaic (i.e., syntheses employing very simple substrates) and modern (i.e., syntheses of compounds with intricate cage structures) aspects of organic chemistry. On the other, it suddenly opened entirely new vistas in chemical research. For example, upstaging sulfur, carbon became the element with the largest number of allotropes. Functionalization of fullerenes gave rise to novel chemical reactions and their products [28], whereas doping with alkali metals produced superconducting fullerites [29]. However, even more tantalizing was the possible existence of entirely new types of substances composed of atoms, ions, or molecules trapped inside fullerene cages.

Since the discovery of the chlorine clathrate by Sir Humphry Davy, 1st Baronet, over 200 years ago [30] and its further characterization by Michael Faraday 12 years later [31], a plethora of investigations on host–guest compounds have been carried out, producing tens of thousands of research papers [32]. The broad spectrum of stabilities of these compounds can be attributed to the diversity of the interactions involved, which include weak chemical bonding, electrostatic effects, London dispersion forces, and steric hindrances. The heights of the resulting barriers to dissociation span the range from a few to tens kcal/mol. In contrast, thanks to the rigidity of the hosts, the guests inside fullerene cages are prevented from escaping by barriers reaching several hundreds of kcal/mol. In addition to their extraordinary stabilities, these species have other unique properties (such as high symmetries and polarizabilities of the hosts) that set them apart from other carceplexes [33], justifying their placement in a separate class of compounds whose name *endohedral complexes* has become part of the common vocabulary of chemists.

The present paper provides (in both scientific and anecdotal manner) a first-hand account on the early electronic structure calculations on endohedral complexes and juxtaposes their predictions against the available experimental data and the results of modern computational work. In addition, it includes a discussion on the prospects for future improvements in accuracy and speed of such calculations. Written as a review, it is intended for a broad audience of chemists, including junior researchers whom it may induce to marvel at the great strides made by quantum chemistry over the last 30 years.

## 2. The Doubters and the Believers

My interest in fullerenes and their derivatives was prompted by a brief conversation that took place in January of 1986. A few weeks after my arrival at Georgetown University to carry out graduate research, my Ph.D. adviser asked me to take a look at the freshly published report on C${}_{60}$[26], commenting that it seemed to belong to the same category as the publications claiming the existence of “polywater” [34] (which upon closer scrutiny turned out to be ordinary sweat [35]). Indeed, on purely entropic grounds, the emergence of highly symmetric C${}_{60}$ cages from a hot soup of carbon oligomers appeared highly implausible even to me, a humble graduate student. On the other hand, the prominence of the m/e=720 peak in the recorded mass spectra could not be easily explained without invoking the notion of a truncated icosahedral structure. It was all very puzzling indeed but I had my research to do and, thus, without giving the subject any substantial thought, I returned to some programming on the IBM AT computer (with CPU running at 8 MHz and 512 kB of RAM) later that day.

The doubts about the veracity of the initial observation of the C${}_{60}$ fullerene did not seem to deter numerous researchers from carrying out a variety of electronic structure calculations on its hypothetical structure [36] that confirmed its high stability. However, a convincing experimental proof of its existence was still missing. In the meantime, a report on the observation of electrolytically induced cold fusion of deuterium was published [37]. Predictably, a plethora of theoretical papers aiming at the explanation of this phenomenon soon appeared [38].

In May of 1989, I joined the faculty of the Chemistry Department of Florida State University (FSU) as an assistant professor. Simultaneously, I was appointed a researcher of the Supercomputer Computations Research Institute (SCRI) located on the top floor of FSU’s Dirac Library building. Founded five years earlier by Joseph Edward Lannutti, SCRI was the first supercomputing center in the US funded by the Department of Energy that supported research within diverse disciplines, such as computer science, physics, chemistry, geology, mathematics, meteorology, and statistics. Although the ETA10 fiasco was unfolding when I arrived at SCRI, the failed machine was soon replaced by Cray Y-MP/432 with four CPUs running UNICOS 5.1 (a version of Unix) at 166 MHz and 256 MB of RAM. Delivering 1.3 GFLOPS peak speed, it was one of the fastest computers in the world at that time. Numerous RISC workstations, such as DECstation 5000/240 and IRIS 4D (each capable of clocking ca. 10 MFLOPS), were also at the disposal of the SCRI researchers. In conjunction with the on-site availability of several system analysts, this hardware made for an excellent computing environment.

## 3. Software Testing and Benchmarking

In late 1989, a new suite of quantum-chemical programs, called TURBOMOLE, appeared [39]. Impressed by the claims of its performance and the fact that it was capable of handling non-Abelian symmetry point groups (a feature that was implemented in the competing software only much later), I requested the pertinent source code from its primary developer Reinhart Ahlrichs [40]. Upon its prompt arrival, it was time to run some benchmarks that would challenge both its advertised performance and the power of the available hardware. After a few hours of deliberations, I suddenly recalled the postulated structure of the C${}_{60}$ cluster. Having a large number of atoms and the icosahedral symmetry, it fit the bill perfectly. Thus, I instructed my post-doctoral associate Eugene D. Fleischmann to optimize its geometry and then, guided by an impulse, I told him to “also throw some simple stuff at the center of it for added fun”.

The calculations running TURBOMOLE on DECstation 5000/240 and IRIS 4D turned out to be unexpectedly fast, obviating the need to engage Cray Y-MP. Constrained by the software and hardware limitations of the 1990s, they involved the Hartree–Fock approximation employed in conjunction with the modest 4-31G (for the carbon atoms of the cage) and DZP (for the F${}^{-}$, Ne, Na${}^{+}$, Mg${}^{2+}$, and Al${}^{3+}$ guests placed at the cage center) basis sets. This level of theory was capable of accounting for the six phenomena that contribute to electronic structures of the studied species, namely: (1) non-zero electrostatic potentials at the cage generated by charged guests, (2) polarization of the cage electrons by these electrostatic potentials, (3) non-zero electrostatic potentials at the guests due to the cage, (4) polarization of the guest electrons by these electrostatic potentials, (5) charge transfers between the guests and the cage, and (6) exchange (closed-shell) interactions between the guests and the cage. However, the stabilizing dispersion (London) interactions between the cage and the guests were completely missing due to the neglect of electron correlation. It should be emphasized that, had those been calculated within the MP2 approximation (which was out of question at that time anyway), their estimates would still have been grossly inaccurate due to the insufficient sizes of basis sets and the inadequacy of the electron correlation treatment at that level of theory.

The computed electronic properties exhibited well pronounced regularities that were later found to be readily explainable (see the next section of this paper). What remained was coining a general name for the hypothetical systems composed of atoms, ions, or molecules encapsulated in fullerene cages. A combination of the Ancient Greek words ἔνδον (inside) and ἕδρα (a face of a geometrical solid) seemed to be an appropriate choice and, thus, the term *endohedral complexes* was introduced in the manuscript we submitted to the Journal of Chemical Physics on 20 August 1990. After the paper was published on 1 March 1991 [41], this term has rapidly gained popularity, the first instance of its subsequent use being the report by Helmut Schwarz and collaborators submitted on 23 May 1991 and published soon thereafter [42]. Needless to say, the true reason (i.e., just testing TURBOMOLE) for carrying out electronic structure calculations on endohedral complexes was not disclosed in our paper.

## 4. Experimental Breakthrough and More Calculations

Unbeknownst to us, just two weeks before submission of our paper, the landmark report on the isolation of macroscopic quantities of the C${}_{60}$ fullerene reached the editorial office of Nature. Its publication on 27 September 1990 [27] once and for all put to rest speculations about this new allotrope of carbon. Although the C${}_{60}$ fullerene now belonged to the realm of reality, the trivial experimental protocol for its production seemed all too easy, the obvious question of “Why has nobody come across it before?” lingering in my mind. This created a dilemma: on one hand, the idea of continuing research on electronic structures of endohedral complexes was quite tempting but, on the other, the embarrassment visited by the “cold fusion” fiasco upon the authors of theoretical publications supporting it was a considerable deterrent. In order to alleviate my worries, I decided to obtain more information from a credible source and thus called Richard Errett Smalley. He must have been quite amused by being questioned on experimental procedures by a junior quantum chemist but nevertheless instantaneously turned me into a believer by suggesting that I acquire a welding power supply, two graphite electrodes, a balloon filled with helium, a water ejector vacuum pump, and a big glass jar (e.g., of the kind used for pickling cucumbers) and then make some fullerenes myself in the garage of my house. Being well aware of my poor manual dexterity, I certainly did not entertain following his advice, starting instead a new series of calculations on the same day.

The results of those calculations, which dealt with the complexes involving several diatomic molecules as the guests, were soon published [43]. Although originally intended for yet another paper [44], the data subsequently obtained at the HF/DZP level of theory for fully optimized geometries of endohedral complexes of the C${}_{60}$ fullerene with H${}^{+}$, Li${}^{+}$, and Na${}^{+}$ were presented in a book chapter [45]. The common thread in the computed properties of these species was the ease with which their values could be explained in a semi-quantitative manner by considering just two phenomena. The first of them is the endohedral effect [43,46], i.e., the presence of an almost constant, positive-valued electrostatic potential inside the fullerene cage. This potential has its origin in the excess of the exohedral negative charge over its endohedral counterpart, which is caused by the curvature of the fullerene cage (to picture this, the reader may consider two extremal cases: that of a flat sheet of carbon atoms, in which the charge is identical on both sides, and that of the unified atom limit of coalesced carbons, in which the entire charge is present outside of the resulting nucleus). The endohedral effect is responsible for the (almost) constant increase in the ionization potential pertaining to the removal of an electron of the guest and the extra stabilization (destabilization) of complexes with negatively (positively) charged guests. The second phenomenon, which is the polarization of the cage electrons by the electrostatic potential of the guest, has multiple effects. In the case of guests that are electrically neutral but polar, it results in the dipole moments of the respective endohedral complexes being given by constant fractions of those of the guests *in vacuo*. Moreover, it promotes enhancement of the dipole moments of the entrapped guests by bond dilatation. In species with charged guests, it provides additional stabilization that is proportional to the squares of their electric charges and is the driving force for their displacements from the cage center. Combined with the changes in the ionization potentials of the cage brought about by the electrostatic potentials of charged guests, these two phenomena also determine the presence/absence of the host-guest charge transfer in endohedral complexes. It is worth noting that the same phenomena are operational in species formed by higher fullerenes, such as C${}_{70}$, C${}_{76}$, C${}_{78}$, C${}_{82}$, and C${}_{84}$ [47].

Another interesting aspect of the guest entrapment that emerged from the aforementioned calculations is the negligible barrier to rotation of small diatomic molecules inside the C${}_{60}$ cage [43,45]. Consequently, solids composed of endohedral complexes involving polar guests are expected to constitute a new class of ferroelectric materials that are distinct from their displacive-type and order–disorder-type counterparts. These endohedral fullerites were predicted [48] to be the first practical realizations of (almost) ideal dipolar lattices, the formation of which is proscribed in the case of ordinary molecular crystals due to the suppression of a free rotation of dipoles brought about by their close packing.

## 5. He@C${}_{60}$: Notation, Isolation, ${}^{3}$He NMR, and the Question of Bonding

Barely one year had passed since the isolation of the C${}_{60}$ fullerene when convincing evidence for species containing metals trapped inside carbon cages was uncovered, prompting the introduction of the “@ symbolism” (attributed to Ori Cheshnovsky) [49]. Pure samples of the particularly stable La@C${}_{82}$ endohedral metallofullerene were obtained two years later (i.e., in September of 1993) [50]. The same month, the He@C${}_{60}$ endohedral complex was finally produced in quantities allowing measurement of the ${}^{3}$He NMR chemical shift of its guest, which turned out to equal −6.3 ± 0.15 [ppm] [51]. The analogous shift of −28.8 ± 0.2 [ppm] was also determined for ${}^{3}$He in the He@C${}_{70}$ complex. Those experimental developments came as a culmination of a year of efforts by Martin Saunders and collaborators that saw a dramatic increase in the helium incorporation fraction from only $\frac{1}{880,000}$[52] to about $\frac{1}{1000}$[53]. Upon learning of those efforts, I rushed the creation of a software chimera composed of the TURBOMOLE core feeding the GIAO-CPHF subroutines (kindly supplied by Peter Pulay) [54]. The resultant theoretical predictions were reported soon thereafter in a paper [55] published in the Journal of the American Chemical Society approximately two months after the disclosure of the experimental work. The ${}^{3}$He chemical shift computed at the GIAO-CPHF/6-31G&DZP level of theory turned out to be quite sensitive to the bonds lengths and angles of the C${}_{60}$ cage, the values of −8.7 and −6.7 [ppm] being obtained at the HF/DZP and MNDO optimized geometries, respectively (note that the latter geometry is much closer to its experimental counterpart). The accuracy of those predictions contrasted with the failure of the simple estimates based upon the London theory [56] that were found to be off by an order of magnitude. In a follow-up paper, the results of the GIAO-CPHF/6-31G&DZP calculations extended to the C${}_{60}^{6+}$, C${}_{70}$, and C${}_{76}$ host cages were presented [57]. Seven years later, in a large collaborative effort, endohedral chemical shifts were computed at the GIAO-CPHF/3-21G level of theory for all the isolated-pentagon, closed-shell (i.e., with non-zero HOMO-LUMO gaps) isomers of the fullerenes comprising between 60 and 86 carbon atoms and for one nonclassical C${}_{72}$ isomer (with the C${}_{2v}$ symmetry) [58]. Those calculations, carried out at the B3LYP/6-31G* optimized geometries, yielded predictions in reasonable agreement with the experimental data. However, the sensitivity of the computed ${}^{3}$He chemical shifts to the geometrical parameters of the cages and the basis sets used in conjunction with the GIAO-CPHF formalism, already well pronounced for the He@C${}_{60}$ and He@C${}_{70}$ species (Table 1), limits the usefulness of such predictions in unambiguous assignment of fullerene structures to the measured ${}^{3}$He NMR spectra of their endohedral complexes.

Before proceeding further, a comment on the issue of the host–guest chemical bonding in endohedral species is in order. In general, the cage critical point (i.e., the minimum) in the electron density of an empty host molecule is replaced by an attractor (i.e., the maximum) upon insertion of the guest atom. Simple topological constraints (i.e., those following from the Poincaré–Hopf index theorem) demand that this alteration of the electron density characteristics is accompanied by the appearance of at least one bond critical point. This observation is readily generalized to the changes in the manifolds of critical points that accompany the formation of complexes with molecules serving as the guests. Indeed, host–guest bond paths are commonly found in electron densities of carceplexes, including those assembled from noble gas atoms and highly symmetric host cages [41,62,63,64]. Thus, for example, there are 30 such paths between the Ne atom and the (nominally) double carbon–carbon bonds of the Ne@C${}_{60}$ endohedral complex [41,62]. The bond points pertaining to these paths give rise to 60 ring points (corresponding to three-membered rings delineated by the Ne atom and the bond points of the double carbon–carbon bonds) and 32 cage points (belonging to 20 cages with triangular and 12 cages with pentagonal bases). Although, thanks to the I${}_{h}$ symmetry of the complex, this manifold of critical points is, without doubt, aesthetically appealing, its presence is dictated entirely by the strictures of mathematical theorems rather than physical or chemical phenomena. For this reason, it cannot be construed as the indication of chemical bonding between the Ne guest and the C${}_{60}$ host. Consequently, a clear distinction has to be maintained between the endohedral complexes discussed in this paper and the endohedral compounds (such as the aforementioned La@C${}_{82}$ endohedral metallofullerene) that involve manifest chemical bonds between the guests and the inner surfaces of the hosts.

## 6. Further Experimental Developments

Although the aforedescribed high-pressure approach to the insertion of the helium atom into the C${}_{60}$ fullerene is readily adaptable to heavier noble gases other than radon [52,53], it produces endohedral complexes in very low yields [65,66,67]. For this reason, it has been superseded by synthetic sequences of cage opening–guest insertion–cage closure called “molecular surgery” [68]. The first endohedral complex obtained in macroscopic quantities via this route was H${}_{2}$@C${}_{60}$[69]. This landmark synthesis, accomplished by Koichi Komatsu, Michihisa Murata, and Yasujiro Murata in 2004, was soon followed by the preparation of He@C${}_{60}$ and He@C${}_{70}$ in 2010 [70], H${}_{2}$O@C${}_{60}$ in 2011 [71], HF@C${}_{60}$ in 2015 [72], CH${}_{4}$@C${}_{60}$ in 2019 [73], Ar@C${}_{60}$ in 2020 [74], Ne@C${}_{60}$ in 2021 [75], and, finally, Kr@C${}_{60}$ in 2022 [76].

The availability of the polar HF@C${}_{60}$ and H${}_{2}$O@C${}_{60}$ species opened an avenue to experimental determination of their properties and, in turn, verification of the pertinent theoretical predictions. Thus, the measured dipole moment of HF@C${}_{60}$ (0.45 ± 0.05 [D] [72]) turned out to be in an excellent agreement with its counterpart (0.43 [D]) originally computed in 1990 at the HF/4-31G&DZP level of theory [43,45]. These values correspond to only 25 ± 3% and 22% of the respective measured and computed dipole moments of the HF molecule *in vacuo*. Interestingly, essentially identical predictions for these absolute and relative dipole moments were obtained with much more sophisticated DF-LMP2/cc-pVTZ calculations carried out almost a quarter of century later [77].

A simple model of the host–guest interactions in endohedral complexes [41,45] predicts the ratio between the dipole moment of a complex and that of its bare guest to be approximately constant and, according to various estimates, equal to between 19 and 28% in the case of the C${}_{60}$ cage [41,43,45,47]. The measured dipole moment of the H${}_{2}$O@C${}_{60}$ species (0.51 ± 0.05 [D] [78] or 0.50 ± 0.05 [D] [79]) amounts to 27 ± 3% of that of the water molecule *in vacuo* and, thus, conforms to this prediction. Although similar magnitudes of this ratio were obtained at various levels of theory, it should be mentioned that (certainly incorrect) reports of its values in excess of 100% also appeared [80]. Per an early theoretical study [48], the combination of a non-zero dipole moment and a low barrier to rotation of the guest should facilitate the formation of a low-temperature (anti)ferroelectric phase of the H${}_{2}$O@C${}_{60}$ fullerite. However, experimental evidence for this phase remains elusive. At the first glance, the observed absence of dielectric anomalies above 8 [K] seemed to be consistent with the predicted transition temperature of ca. 4 [K] [81]. Alas, no peak-like anomaly in the measured heat capacity was subsequently detected all the way down to 0.6 [K] [82]. Although cryogenic INS and NMR experiments revealed a splitting of the triply degenerate ground state of ortho-water trapped in the C${}_{60}$ cage that was speculated to reflect cooperative electric dipole alignment [83], another mechanism behind this phenomenon appears to be much more likely on the basis of a thorough theoretical analysis [84]. The reasons for the failure to uncover the predicted ferroelectric phases of the HF@C${}_{60}$and H${}_{2}$O@C${}_{60}$ fullerites remain obscure thus far.

In contrast to its reasonable predictions of dipole moments and the extents of their endohedral screening, the Hartree–Fock approximation fails to account for the red shifts in the wavenumbers of fundamental vibrational transitions observed upon the encapsulation of small molecules in the C${}_{60}$ fullerene. Thus, blue shifts were computed at the HF/4-31G&DZP level of theory for the H${}_{2}$, N${}_{2}$, CO, HF, and LiH guests, the red shift being obtained only for LiF [43,45]. These predictions turned out to be in variance with the red shifts of 98.8 and 170.5 [cm${}^{-1}$] measured, respectively, for the H${}_{2}$[85] and HF [72] guests. The latter value was accurately reproduced by the (scaled) red shift of 174 [cm${}^{-1}$] afforded by calculations at the DF-LMP2/cc-pVTZ level of theory [77]. In the case of the H${}_{2}$ guest, a shift of 119 [cm${}^{-1}$] was predicted, which amounts to overestimation by ca. 20%. Interestingly, the red shift of 96 [cm${}^{-1}$] computed for the LiF guest turned out to match closely its aforementioned HF/4-31G&DZP counterpart of 97.4 [cm${}^{-1}$].

The large errors in the IR shifts calculated within the Hartree–Fock approximation stem from the neglect of electron correlation effects, especially the stabilizing dispersion (London) interactions between the cage and the guests. Requiring high levels of theory for accurate reproduction, these effects are not reliably accounted for by the presently available approximate functionals of the KS-DFT formalism. For example, although a surprisingly accurate estimate of 172.3 [cm${}^{-1}$] was obtained at the MN15/cc-pVTZ level of theory for the red shift of HF, its H${}_{2}$ counterpart of 142.3 [cm${}^{-1}$] turned out to be far less satisfactory [86]. Ever worse, the same type of calculation predicted a significant (0.189 [Å]) displacement of the He guest from the center of the C${}_{60}$ cage that is ruled out by direct experimental evidence [87].

Alkali metal cations trapped inside fullerene cages comprise a distinct class of endohedral complexes. The first representative of this class, namely Li${}^{+}$@C${}_{60}$, was isolated in the form of its [Li${}^{+}$@C${}_{60}$][SbCl${}_{6}^{-}$] salt by Hiroshi Sawa, Hiromi Tobita, and their collaborators in 2009 [88]. An inventive process involving simultaneous depositions of C${}_{60}$ and the Li plasma followed by chemical oxidation of the produced Li@C${}_{60}$ endohedral complex [88,89,90] was employed in the synthesis of that salt and its analogs, such as [Li${}^{+}$@C${}_{60}$][PF${}_{6}^{-}$] [90,91,92]. Single-crystal X-ray diffraction experiments revealed that at 370 [K] the lithium cation in [Li${}^{+}$@C${}_{60}$][SbCl${}_{6}^{-}$] is displaced by 1.34 [Å] from the cage center [88]. In the case of [Li${}^{+}$@C${}_{60}$][PF${}_{6}^{-}$], the measured diffraction patterns were consistent with a positional disordering of the cation within a shell with a radius of about 1.5 [Å] at 155 [K] and the presence of two crystallographically equivalent positions of Li${}^{+}$ located 1.40 ± 0.01 [Å] from the cage center at 22 [K] [91]. These experimental findings are in agreement with the original HF/DZP prediction of the radial displacement amounting to 1.30 [Å] [45] and the displacements of 1.40, 1.43, and 1.50 [Å] subsequently computed at, respectively, the M06-2X/6-31G** [93], MN15/cc-pVTZ [86], and B3LYP/cc-pVTZ [94] levels of theory.

According to the already discussed simple model [41,45], the host–guest interaction in the Li${}^{+}$@C${}_{60}$ endohedral complex has a negligible covalent component. This assertion is supported by several experimental observations. First of all, the radial displacement of the guest exhibits very weak angular dependence that is reflected in both the aforementioned positional disordering of the cation within a shell in the [Li${}^{+}$@C${}_{60}$][PF${}_{6}^{-}$] salt at 155 [K] [91] and the presence of a single peak in its ${}^{13}$C NMR spectrum of its solutions [90]. Second, its small (0.60 [ppm] [88] or 0.63 [ppm] [90]) upfield shift relative to C${}_{60}$ is consistent with the absence of both covalent host–guest interactions and a significant angular variation of the electrostatic potential generated within the host cage by the charged guest. The resulting uniform shift in the orbital energies of the host is confirmed by the hardly noticeable differences among the UV/VIS spectra of [Li${}^{+}$@C${}_{60}$][SbCl${}_{6}^{-}$], [Li${}^{+}$@C${}_{60}$][PF${}_{6}^{-}$], and the pristine C${}_{60}$[88,90]. This shift is also consistent with the results of cyclic voltammetry measurements [88,90], though the observed differences between the reduction potentials of Li${}^{+}$@C${}_{60}$ and C${}_{60}$ are much smaller than those predicted from the corresponding electron affinities *in vacuo* due to the solvation effects that are far more pronounced for the former species.

In contrast to those of the H${}_{2}$O@C${}_{60}$ endohedral complex, crystals of the [Li${}^{+}$@C${}_{60}$][PF${}_{6}^{-}$] salt undergo a low-temperature transition to an antiferroelectric phase. The measured critical temperature of this transition equals 24 [K] [92]. It should be noted that displacing the lithium cation from the cage center by ca. 1.4 [Å] generates the dipole moment of ca. 6.7 [D], which corresponds to the theoretically predicted [48] critical temperature of ca. 28 [K]. Unless fortuitous, the accuracy of this prediction seems to underscore the minor role played by the PF${}_{6}^{-}$ counterions in the formation of the dipole-ordered phase.

## 7. Standing the Test of Time

As one may conclude from the long list of comparisons between the theoretical and experimental data presented above, the pioneering electronic structure calculations on endohedral complexes [41,43,45] have a mixed scorecard on accuracy of their predictions. Cutting-edge by the standards prevalent 30 years ago and low-level when juxtaposed against their modern counterparts, those calculations yielded properties of endohedral complexes that fall into four broad categories, namely: (1) those in agreement with experimental results and the predictions of more sophisticated theoretical approaches, (2) those in disagreement with experimental results that are nevertheless accurately reproduced by calculations involving higher levels of theory, (3) those for which reliable theoretical predictions are not available thus far, and (4) those still awaiting experimental verification. Although the boundaries between these categories are somewhat fuzzy due to the imprecise nature of the term ”in agreement”, such a classification of the computed properties is attempted below.

The first of the above categories encompasses the majority of the predictions afforded by the simple model of the host-guest interactions [41,45] that emerged from the early electronic structure calculations. Indeed, the host cages of the endohedral complexes turn out to have geometries virtually identical with those of the empty C${}_{60}$ fullerene, their orbitals remaining unchanged (by neutral guests) or being uniformly shifted (by charged guests) as evidenced by the insensitivity of their UV/VIS spectra to the insertion of the guest. As predicted, the charged guests are found to displace from the cage center, whereas the dipole moments of the polar ones are reduced by a constant factor. The radial displacements and the endohedral screenings have the measured values that are close to their computed counterparts. Small diatomic guests (H${}_{2}$, N${}_{2}$, CO, HF, LiH, and LiF) rotate almost freely inside the C${}_{60}$ cage, whereas the Li${}^{+}$ cation barely exhibits any angular preference in its radial displacement. As expected, none of the endohedral complexes formed with these guests involves the host-guest charge transfer. On the other hand, inserting the ionization potential I(G)=5.4 [eV] of the lithium atom and its charge Q(G)=0 into the pertinent stability condition I(G)−7.0−6.1Q(G)>0[45] leads to the conclusion that the complex with the nominal composition of Li@C${}_{60}$ has the actual electronic structure of Li${}^{+}$@C${}_{60}^{-}$. This prediction is unequivocally supported by the available experimental data [89].

The vibrational frequencies of the guests can be ascribed to the second of the above categories. Their computed values are strongly affected by electron correlation effects whose accurate reproduction requires high levels of theory (certainly going beyond the DFT treatment). However, even the sophisticated DF-LMP2/cc-pVTZ calculations turn out to overestimate by 20% the red shift in the wavenumber of the fundamental vibrational transition effected by the insertion of the H${}_{2}$ molecule into the C${}_{60}$ fullerene cage. Surprisingly, these calculation predict the red shift for the LiF guest that is very close to its counterpart originally obtained at the HF/4-31G&DZP level of theory. These observations may imply that in fact the vibrational frequencies of the guests belong to the third category of the computed properties.

Inspection of Table 2 convincingly reveals that the complexation energies, defined as the differences between the energies of the endohedral complexes and those of their constituents, are indisputable members of that category. To begin with, the Hartree–Fock approximation substantially underestimates stabilities of the endohedral complexes of fullerenes [41,43,45,61,95,96,97,98]. At first glance, it may seem that this serious deficiency could be readily rectified or at least alleviated by augmenting the simple model of the host–guest interactions mentioned several times in this paper with an approximate energy term describing the dispersion (London) interactions that are missing at the uncorrelated levels of theory. However, the published attempts in that direction [96,99] were found under closer scrutiny to produce unsatisfactory results [100]. The same is true about diverse implementations of the KS-DFT formalism that, depending on the functional employed, yield complexation energies that either grossly underestimate [86,93] or overestimate [95,101,102,103,104] their counterparts obtained with rigorous approaches to the electron correlation problem, putting the reliability of the DFT-based calculations in doubt. Although various variants of the MP2 theory fare better in this respect [61,77,96,98,99,101,104,105,106], they do not produce sufficiently convergent results, as illustrated by the case of the LiF guest for which the complexation energy computed at the MP2/cc-pVTZ level of theory (−22.8 [kcal/mol] [77]) is much closer to its HF/4-31G&DZP counterpart of −14.9 [kcal/mol] [43,45] than to that of −50.6 [kcal/mol] obtained from the extrapolation involving the RI-MP2/def2-TZVPP and RI-MP2/def2-QZVPP energies [99]. Some of these discrepancies stem from obvious errors in computations and/or reporting of the data (see, e.g., one of the sets of the MP2/6-31G** energies listed in Table 2). However, more clues to their origins are provided by the examination of two sets of the complexation energies calculated at the MP2/cc-pVTZ level of theory and its variant employing density fitting (i.e., DF-LMP2/cc-pVTZ), namely those of −15.5 (DF-LMP2/cc-pVTZ), −21.6 (MP2/cc-pVTZ), and −16.8 [kcal/mol] (BSSE-corrected MP2/cc-pVTZ) for the N${}_{2}$@C${}_{60}$ species and those of −24.5, −32.8, and −22.8 [kcal/mol] for LiF@C${}_{60}$ [77]. In both cases, the basis-set-superposition error (BSSE) is quite prominent, amounting to between 29 and 44% of the corrected MP2/cc-pVTZ energies despite the rather large basis set being employed. The comparable differences between the uncorrected MP2/cc-pVTZ and DF-LMP2/cc-pVTZ energies arise from the close similarity of the latter to their BSSE-corrected MP2/cc-pVTZ counterparts. It is tempting to attribute this similarity to the assertion that “local correlation methods by design significantly reduce the influence of BSSE on the resulting interaction energies” [77], which is, alas, invalidated by the comparison of the published uncorrected and BSSE-corrected DLPNO-CCSD(T)/def2-TZVP complexation energies of Na${}^{+}$@C${}_{60}$ (−22.4 vs. −14.6 [kcal/mol]) and CH${}_{4}$@C${}_{60}$ (−15.3 vs. −11.1 [kcal/mol]) [86]. Thus, it is clear that further improvements in the accuracy of the predictions concerning stabilities of the endohedral complexes of fullerenes hinge upon (admittedly costly) employment of even larger basis sets (likely in conjunction with extrapolation schemes) and tightening of the density-fitting tolerances. Only under such circumstances, the chemical accuracy (i.e., that of ca. 1 [kcal/mol]) can be at least approached, the computed complexation energies being of true benchmark quality (which, despite the claims to the contrary, is not attained by the recent DLPNO-CCSD(T)/def2-TZVP data [86] that, even in the case of He@C${}_{60}$, deviate by as much as 3.6 [kcal/mol] from those obtained at the DLPNO-CCSD(T)/cc-pV(T&Q)Z level of theory [100]).

The ${}^{3}$He chemical shifts in the NMR spectra of the endohedral complexes involving the helium atom as the guest provide another example of the properties for which theoretical predictions have not achieved satisfactory accuracy thus far. It is unclear at present whether the errors in the computed values of these shifts are due to the neglect of correlation effects within the GIAO-CPHF formalism or their high sensitivity to the geometrical parameters of the host cages employed in the calculations.

Finally, a few words are in order concerning the phenomena for which theoretical predictions are available while experimental data are still lacking. The first of them is the endohedral effect, i.e., the constant positive-valued shift in the ionization energies of the guests effected by their insertion into the host cages. Obviously, experimental observation of this phenomenon requires the first ionization energy of the guest being smaller than that of the host. In the case of the C${}_{60}$ fullerene, this means I(G)≤7.6 [eV] which is barely above the threshold of I(G)=7.0 [eV] imposed by the aforementioned stability condition I(G)−7.0−6.1Q(G)>0 [45] for electrically neutral [Q(G) = 0] guests. Another phenomenon not readily amenable to experimental verification is the influence of the encapsulation upon the equilibrium geometries of the guests, which, in the case of several diatomic molecules, amounts to the predicted changes of, at most, 0.08 [Å] in their bond lengths [43,45,77]. The relative smallness of these changes, coupled with the positional disorder of the guests, precludes their precise measurement by means of the single-crystal X-ray diffraction. On the other hand, the barriers to rotation of the guests (which couple their motions to those of the host cages) render the determination of their geometries from the measured rovibrational spectra impossible.

The blunt message that emerges from the above considerations is the following: the “easy” properties of endohedral complexes (including the repeatedly rediscovered [109] screening of the dipole moments of the guests) have already been properly reproduced by the bare-bones calculations carried out in the 1990s, whereas the “tough” ones are still awaiting reliable computations 30 years later.

## 8. Future Prospects

In 1990, it took ca. 10 days to complete (on the DECstation 5000/240) a HF/4-31G&DZP calculation on an endohedral complex of the C${}_{60}$ fullerene with a heteronuclear diatomic molecule [43]. In 2014, an analogous DF-LMP2/cc-pVTZ calculation required between 2 and 3 days on a computing node with four Intel Xeon^®^ X5650@2.67GHz processors [77]. This comparison vividly illustrates the progress in both the quantum-chemical software and the computing hardware that has been taking place at the turn of the twentieth century. The expected continuation of this progress bodes well for the feasibility of electronic structure calculations that have to be carried out in order to bring the accuracy of the predictions concerning various properties of the endohedral complexes of fullerenes to the level desired by both theoretical and experimental chemists.

The first issue to be addressed by these calculations is that of highly accurate geometries of the fullerene cages. Except for those of C${}_{60}$ and C${}_{70}$, these geometries cannot be determined in the gas phase, whereas X-ray diffraction measurements produce data affected by the crystal-packing effects. The availability of the geometrical parameters pertaining to isolated cages is of crucial importance to reliable predictions of the ${}^{3}$He chemical shifts in the NMR spectra of the helium-containing endohedral complexes whose computed values are known to be remarkably sensitive to the cage geometries employed in their calculations (see Table 1). Although the actual values of these parameters are barely affected by solvent effects and/or the insertion of the guest, their accurate computation is contingent upon proper treatment of electron correlation. Geometry optimizations at the DLPNO-CCSD/cc-pVTZ level of theory, which most probably suffices for this purpose, are feasible (though admittedly very expensive) with the hardware available at modestly equipped computer centers.

The next set of calculations worth performing is that of the aforementioned ${}^{3}$He chemical shifts. Initially, in order to assess the dependence of the computed shifts on the basis sets employed and explore the possibility of their extrapolation to the respective complete-basis-set (CBS) limits, the values of NICS at the cage centers should be obtained at the aforedescribed benchmark geometries within the Hartree–Fock approximation. In the case of the resulting GIAO-CPHF shifts turning out to deviate significantly from their observed counterparts, attempts should be made to repeat these calculations within the GIAO-MP2 formalism (which is now feasible thanks to the very recent developments [110]). Upon identification of the proper level of theory, at which the convergence of the computed NICSs to sufficiently accurate values is attained, the final round of calculations involving the encapsulated guest should be carried out (most likely without the need for reoptimization of the cage geometries).

The aforediscussed discrepancies in the computed complexation energies that persist even at the highest levels of theory employed thus far call for thorough investigations. First of all, the geometries of the endohedral complexes in question should be fully reoptimized at the same level of theory at which the energies are calculated. The resources required for carrying out such reoptimizations are within reach of many researchers. The energy components that are affected the most by the resulting geometry changes should be identified and, on the basis of this information, the level of theory representing a balanced trade-off between the computational cost and the accuracy of the computed data should be selected for extended calculations on the endohedral complexes involving other guests. Attention should also be paid to the prediction of the shifts in the wavenumbers of fundamental vibrational transitions observed upon the encapsulation of the guest, whose present accuracy leaves much room for improvement. The computation of these shifts may require (at least approximate) treatment of vibrational anharmonicities.

The scarcity of the data pertaining to endohedral complexes involving fullerenes other than C${}_{60}$ should also be addressed. Such species are expected to exhibit several properties of both practical and theoretical importance. For example, the endohedral motions of the Na${}^{+}$ cation inside capped single-walled carbon nanotubes should give rise to a multitude of observable transitions in the far IR region, making such materials potentially useful as components of radar-absorbing coatings [111].

## 9. Some Last Words

In 1900, the feasibility of synthesizing organic substances from simple inorganic precursors was widely accepted thanks to the experimental work of Gmelin carried out 75 years earlier. The carbon arc light was one of the many inventions of the Victorian era. Helium gas was scarce but certainly procurable by researchers. Vacuum distillation requiring pressures as low as 100 [Torr] was routinely carried out in laboratories of organic chemists. Thus, all the technology and all the materials required for the production of fullerenes were available. Alas, this production has not materialized for another 90 years.

One can only speculate on the nature of the hindrance at play. However, one may venture that the idea of a carbon allotrope consisting of “buckyballs”, which appeared doubtful to many even in 1985, would have been regarded as utterly absurd in 1900. At that time, many concepts of organic chemistry and classes of organic compounds that we now take for granted were still unknown. First of all, the precise meaning of aromaticity was poorly understood. Second, neither cage hydrocarbons nor polycyclic aromatic hydrocarbons with more than a few rings were either isolated or synthesized. In light of these facts, one can readily picture the wine-red tint acquired by an organic solvent used for cleaning a glassware casing of some carbon arc experiment being dismissed as coming from contaminants, tar, or simply dirt. Thus, all in all, it is very probable that the main reason for the (seemingly difficult to understand) delay in the discovery of fullerenes was simply the lack of imaginative speculation that would only later become unlocked thanks to the accumulated knowledge brought by many decades of scientific investigations carried out in the twentieth century.

The history of research on fullerenes and their endohedral complexes provides an interesting example of interactions among organic, physical, and theoretical chemists in which the elements of collaboration, competition, and pure serendipity contributed to the emergence of an entirely new class of substances with entirely new properties. This observation is particularly pertinent to the endohedral complexes that were first discovered in silico and only then *in vacuo* and in vitro. As the rate of progress in the efficiency of quantum-chemical software and in the performance of the hardware it runs on greatly exceeds that in experimental techniques, electronic structure calculations are slated to play an increasingly important role in research on those fascinating species. One expects these scientific endeavors being facilitated by the synergy between mathematical rigor and imaginative speculation.

## Figures and Tables

**Table 1:** The dependence of the computed ${}^{3}$He chemical shifts [ppm] in the He@C${}_{60}$ and He@C${}_{70}$ endohedral complexes on the level of theory ${}^{\left(a\right)}$.

GIAO-CPHF Basis Set	Geometry	The Host	
		C${}_{60}$	C${}_{70}$
6-31G&DZP [55,57]	MNDO	−6.7	−17.3
	HF/DZP	−8.7	
3-21G [58]	B3LYP/6-31G*	−8.0	−23.1
DZ&TZP [59,60]	MNDO	−11.7	−23.0
	HF/3-21G	−12.9	−24.0
	BP86/3-21G	−11.0	−25.2
DZP&TZP [58,59]	BP86/3-21G	−11.1	−28.4
	BP86/TZP	−10.3	−29.2
	MP2/TZP	−8.5	−31.1
6-31G** [61]	MP2/TZP	−7.1	

^(*a*)^ The differences between the calculated isotropic chemical shieldings of the helium atom in the endohedral complex and *in vacuo* are listed.

**Table 2:** The complexation energies [kcal/mol] of the X@C${}_{60}$ species.

Level of Theory ${}^{\left(a\right)}$	X									
	He	Ne	Ar	Li${}^{+}$	Na${}^{+}$	H${}_{2}$	HF	LiH	LiF	N${}_{2}$
HF/4-31G&DZP [41,43,45]		0.4			−7.5 ${}^{\left(b\right)}$	1.2	−2.0	−2.4	−14.9	9.6
HF/DZP [45]	0.3	0.5	5.2	−11.6 ${}^{\left(c\right)}$	−4.2 ${}^{\left(d\right)}$					
HF/6-31G* [95]	3.2	1.7	9.3							
HF/def2-TZVPP [96]	0.6	1.5	9.2							
HF/6-31G** [61]	1.0	1.4	8.8							
**HF/3-21G [97]**				−9.4 ${}^{\left(e\right)}$	−8.8 ${}^{\left(e\right)}$					
HF/6-31G [98]							−2.5			
B3LYP/6-31G** [101,102]	1.4	0.9	8.4	−1.0	−0.9					
B3LYP/6-311G* [103]									−9.8	
B3LYP/6-311G(2d,2p) [104]		−3.0				1.3				7.9
wB97XD/6-31G* [95]	−1.7	−4.1	−8.6							
M06-2X/Def2-TZVP [107]		−5.1			−16.3		−12.5			
M06-2X/6-31G** [93]					−25.8					
**MN15/cc-pVTZ [86]**	−4.4	−9.0	−16.4	−28.6	−19.7	−10.7	−16.6			
MN15/aug-cc-pVDZ [108]							−14.9			
**MPWB1K/6-311G(2d,2p) [104]**		−3.8				−7.2				−9.5
PBE-D/aug-def2-TZVPP [106]						−5.6				
B2PLYP-D/QZVP [106]						−6.6				
MP2/6-31G [98]							−7.5			
MP2/6-31G** [61,104]	−0.3	−1.9	−2.5			−2.4				−8.9
MP2/6-31G** [101]	0.6	1.3	9.2							
**MP2/6-311G(2d,2p) [104]**						−6.1				−15.5
MP2/(d,p)-6-311G** [104]						−6.9				
**MP2/TZP&cc-pVTZ [61]**	−2.0									
**MP2/cc-pVTZ [77]**						−6.4	−8.6	−24.3	−22.8	−16.8
MP2/aug-def2-TZVPP [106]						−9.5				
RI-MP2/def2-TZVPP [96,99]	−1.8	−3.4	−17.0							
RI-MP2/def2-QZVPP [99]	−2.3	−4.5	−18.6							
RI-MP2/CBS ${}^{\left(f\right)}$ [99]	−2.6	−5.4	−19.5				−11.8		−50.6	
**SCS-MP2/6-311G(2d,2p) [104]**						−4.2				−8.6
SCS-MP2/aug-def2-TZVPP [106]						−7.4				
RI-SCS-MP2/def2-TZVPP [99]	−1.2	−2.2	−11.0							
RI-SCS-MP2/def2-QZVPP [99]	−1.6	−3.1	−12.2							
RI-SCS-MP2/CBS ${}^{\left(g\right)}$ [99]	−1.9	−3.8	−12.9				−8.4		−33.6	
DF-LMP2/cc-pVTZ [105]							−6.9			
**DLPNO-CCSD(T)/def2-TZVP [86]**	−1.4	−3.1	−12.2	−22.2	−14.6	−5.9	−10.4			
**DLPNO-CCSD(T)/cc-pV(T&Q)Z [100]**	−5.0	−6.6	−12.3							

^(*a*)^ The levels of theory corresponding to the BSSE-corrected data listed in boldface. ^(*b*)^ −6.7 [kcal/mol] for Na+ at the cage center. ^(*c*)^ −4.4 [kcal/mol] for Li+ at the cage center. ^(*d*)^ −3.8 [kcal/mol] for Na+ at the cage center. ^(*e*)^ Values for Li+ and Na+ at the cage center. ^(*f*)^ Extrapolated from the RI-MP2/def2-TZVPP and RI-MP2/def2-QZVPP data. ^(*g*)^ Extrapolated from the RI-SCS-MP2/def2-TZVPP and RI-SCS-MP2/def2-QZVPP data.

## Data Availability

Not applicable.

## References

[1] Gmelin L. (1825). Ueber einige merkwürdige, bei der Darstellung des Kaliums nach der Brunner’schen Methode, erhaltene Substanzen. Ann. Phys. Chem..

[2] Liebig J. (1834). Ueber das Verhalten des Kohlenoxyds zu Kalium. Ann. Chem. Pharm..

[3] Nietzki R., Benckiser T. (1885). Ueber Hexaoxybenzolderivate und ihre Beziehungen zur Krokonsäure und Rhodizonsäure. Berichte Dtsch. Chem. Ges..

[4] Wöhler F. (1828). Ueber künstliche Bildung des Harnstoffs. Ann. Phys. Chem..

[5] Faraday M. (1825). On new compounds of carbon and hydrogen, and on certain other products obtained during the decomposition of oil by heat. Philos. Trans. R. Soc..

[6] Van’t Hoff J.H. (1874). Sur les formules de structure dans l’espace. Arch. Néerl. Sci. Exact. Nat..

[7] Le Bel J.A. (1874). Sur les relations qui existent entre les formules atomiques des corps organiques et le pouvoir rotatoire de leurs dissolutions. Bull. Soc. Chim. Fr..

[8] Baeyer A. (1885). Ueber Polyacetylenverbindungen (Zweite Mittheilung). Ber. Dtsch. Chem. Ges..

[9] Willstätter R., Waser E. (1911). Über Cyclo-octatetraen. Ber. Dtsch. Chem. Ges..

[10] Diederich F., Staab H.A. (1978). Benzenoidversus Annulenoid Aromaticity: Synthesis and Properties of Kekulene. Angew. Chem. Int. Ed. Engl..

[11] Martin R. (1974). H . The Helicenes. Angew. Chem. Int. Ed. Engl..

[12] Wiberg K.B., Lampman G.M., Ciula R.P., Connor D.S., Schertler P., Lavanish J. (1965). Bicyclo[1.1.0]butane. Tetrahedron.

[13] Wiberg K.B., Walker F.H. (1982). [1.1.1]Propellane. J. Am. Chem. Soc..

[14] Katz T.J., Acton N. (1973). Synthesis of Prismane. J. Am. Chem. Soc..

[15] Whitlock H.W. (1962). Tricyclo[4.4.0.03.8]Decane. J. Am. Chem. Soc..

[16] Prelog V., Seiwerth R. (1941). Über die Synthese des Adamantans. Berichte.

[17] Tobler H., Klaus R.O., Ganter C. (1975). Wurtzitan (Tetracyclo[5.3.1.12,6.04,9]dodecan). Helv. Chim. Acta.

[18] Hamon D.P.G., Taylor G.F. (1976). A synthesis of tetracyclo[5,3,1,12,6,04,9]dodecane (iceane). Aust. J. Chem..

[19] Cupas C., Schleyer P.v.R., Trecker D.J. (1965). Congressane. J. Am. Chem. Soc..

[20] Eaton P.E., Cole T.W. (1964). Cubane. J. Am. Chem. Soc..

[21] Ternansky R.J., Balogh D.W., Paquette L.A. (1982). Dodecahedrane. J. Am. Chem. Soc..

[22] Wasserman E. (1960). The Preparation of Interlocking Rings: A Catenane. J. Am. Chem. Soc..

[23] Schill G., Lüttringhaus A. (1964). The Preparation of Catena Compounds by Directed Synthesis. Angew. Chem. Int. Ed. Engl..

[24] Pedersen C.J. (1967). Cyclic polyethers and their complexes with metal salts. J. Am. Chem. Soc..

[25] Dietrich B., Lehn J.M., Sauvage J.P. (1969). Les Cryptates. Tetrahedron Lett..

[26] Kroto H.W., Heath J.R., O’Brien S.C., Curl R.F., Smalley R.E. (1985). C_60_: Buckminsterfullerene. Nature.

[27] Krätschmer W., Lamb L.D., Fostiropoulos K., Huffman D.R. (1990). Solid C_60_: A new form of carbon. Nature.

[28] Hirsch A., Brettreich M. (2006). Fullerenes: Chemistry and Reactions.

[29] Hebard A.F., Rosseinsky M.J., Haddon R.C., Murphy D.W., Glarum S.H., Palstra T.T.M., Ramirez A.P., Kartan A.R. (1991). Superconductivity at 18 K in potassium-doped C_60_. Nature.

[30] Davy H. (1811). On a combination of oxymuriatic gas and oxygene gas. Philos. Trans. R. Soc..

[31] Faraday M. (1823). On Hydrate of Chlorine. Quart. J. Sci. Lit. Arts.

[32] The search in Google Scholar for “inclusion compounds” alone returns over 30,000 entries.

[33] Jasat A., Sherman J.C. (1999). Carceplexes and Hemicarceplexes. Chem. Rev..

[34] Derjaguin B. (1983). Polywater reviewed. Nature.

[35] Rousseau D.L. (1971). “Polywater” and Sweat: Similarities between the Infrared Spectra. Science.

[36] Cioslowski J. (1995). Electronic Structure Calculations on Fullerenes and Their Derivatives.

[37] Fleischmann M., Pons S. (1989). Electrochemically induced nuclear fusion of deuterium. J. Electroanal. Chem..

[38] Chechin V.A., Tsarev V.A., Rabinowitz M., Kim Y.E. (1994). Critical Review of Theoretical Models for Anomalous Effects in Deuterated Metals. Int. J. Theor. Phys..

[39] Ahlrichs R., Bär M., Häser M., Hom H., Kölmel C. (1989). Electronic Structure Calculations on Workstation Computers: The Program System Turbomole. Chem. Phys. Lett..

[40] Having this code handy was a mixed blessing. On one hand, it allowed tinkering with the program necessary for extraction of some data not available from standard output. On the other, it caused some troubles when I instructed a FORTRAN-proficient undergraduate student to fix the sluggish convergence of geometry optimization in her calculations. To her chagrin (and my surprise), it turned out that the controlling variable (i.e., the shift in the Hessian eigenvalues) was named with an English word that upon changing just one vowel becomes its German (equally vulgar) equivalent.

[41] Cioslowski J., Fleischmann E.D. (1991). Endohedral Complexes: Atoms and Ions Inside the C_60_ Cage. J. Chem. Phys..

[42] Weiske T., Bohme D.K., Hrušák J., Krutschmer W., Schwarz H. (1991). Endohedral Cluster Compounds: Inclusion of Helium within C60•⊕ and C70•⊕ through Collision Experiments. Angew. Chem. Int. Ed. Engl..

[43] Cioslowski J. (1991). Endohedral Chemistry: Electronic Structures of Molecules Trapped Inside the C_60_ Cage. J. Am. Chem. Soc..

[44] These data were included in a manuscript submitted to one of the leading chemical journals. The expected paper was later cited (as “in press”) in a survey of electronic structure calculations on endohedral complexes intended for a book chapter, in which all the data obtained thus far were compiled. Under normal circumstances, the book would appear much later than the journal article. However, the editor of the journal dragged his feet with such efficacy that the book was published about the time the manuscript was going to be finally accepted. Noticing this, he happily pronounced the manuscript being “prior publication” and summarily rejected it. The dummy citation has remained in the book chapter.

[45] Cioslowski J., Davies J.E.D. (1992). Spectroscopic and Computational Studies of Supramolecular Systems.

[46] Cioslowski J., Nanayakkara A. (1992). Endohedral Effect in Inclusion Complexes of the C_60_ Cluster. J. Chem. Phys..

[47] Cioslowski J., Raghavachari K. (1993). Electrostatic Potential, Polarization, Shielding, and Charge Transfer in Endohedral Complexes of the C_60_, C_70_, C_76_, C_78_, C_82_, and C_84_ Clusters. J. Chem. Phys..

[48] Cioslowski J., Nanayakkara A. (1992). Endohedral Fullerites: A New Class of Ferroelectric Materials. Phys. Rev. Lett..

[49] Chai Y., Cuo T., Jin C., Haufler R.E., Chibante L.P.F., Fure J., Wang L., Alford J.M., Smalley R.E. (1991). Fullerenes with Metals Inside. J. Phys. Chem..

[50] Kikuchi K., Suzuki S., Nakao Y., Nakahara N., Wakabayashi T., Shiromaru H., Saito K., Ikemoto I., Achiba Y. (1993). Isolation and Characterization of the Metallofullerene LaC_82_. Chem. Phys. Lett..

[51] Saunders M., Jimenez-Vazquez H.A., Cross R.J., Mroczkowski S., Anet F.A.L., Freedberg D.I. (1994). Probing the Interior of Fullerenes by ^3^He NMR Spectroscopy of Endohedral ^3^He@C_60_ and ^3^He@C_70_. Nature.

[52] Saunders M., Jimenez-Vazquez H.A., Cross R.J., Poreda R.J. (1993). Stable Compounds of Helium and Neon: He@C_60_ and Ne@C_60_. Science.

[53] Saunders M., Jimenez-Vazquez H.A., Cross R.J., Mroczkowski S., Gross M.L., Giblin D.E., Poreda R.J. (1994). Incorporation of Helium, Neon, Argon, Krypton, and Xenon into Fullerenes using High Pressure. J. Am. Chem. Soc..

[54] Wolinski K., Hinton J.F., Pulay P. (1990). Efficient Implementation of the Gauge-Independent Atomic Orbital Method for NMR Chemical Shift Calculations. J. Am. Chem. Soc..

[55] Cioslowski J. (1994). Endohedral Magnetic Shielding in the C_60_ Cluster. J. Am. Chem. Soc..

[56] Elser V., Haddon R.C. (1987). Icosahedral C_60_: An aromatic molecule with a vanishingly small ring current magnetic susceptibility. Nature.

[57] Cioslowski J. (1994). Endohedral magnetic shielding in fullerenes. A GIAO CPHF study. Chem. Phys. Lett..

[58] Chen Z., Cioslowski J., Rao N., Moncrieff D., Buehl M., Hirsch A., Thiel W. (2001). Endohedral chemical shifts in higher fullerenes with 72–86 carbon atoms. Theor. Chem. Acc..

[59] Bühl M., Wüllen C.V. (1995). Computational evidence for a new C_84_ isomer. Chem. Phys. Lett..

[60] Bühl M., Thiel W., Jiao H., Schleyer P.v.R., Saunders M., Anet F.A.L. (1994). Helium and Lithium NMR Chemical Shifts of Endohedral Fullerene Compounds: An ab Initio Study. J. Am. Chem. Soc..

[61] Bühl M., Patchkovskii S., Thiel W. (1997). Interaction energies and NMR chemical shifts of noble gases in C_60_. Chem. Phys. Lett..

[62] Cioslowski J., Mixon S.T., Edwards W.D. (1991). Weak Bonds in the Topological Theory of Atoms in Molecules. J. Am. Chem. Soc..

[63] Haaland A., Shorokhov D.J., Tverdova N.V. (2004). Topological Analysis of Electron Densities: Is the Presence of an Atomic Interaction Line in an Equilibrium Geometry a Sufficient Condition for the Existence of a Chemical Bond?. Chem. Eur. J..

[64] Cerpa E., Krapp A., Vela A., Merino G. (2008). The Implications of Symmetry of the External Potential on Bond Paths. Chem. Eur. J..

[65] DiCamillo B.A., Hettich R.L., Guiochon G., Compton R.N., Saunders M., Jimenez-Vazquez H.A., Khong A., Cross R.J. (1996). Enrichment and characterization of a noble gas fullerene: Ar@C_60_. J. Phys. Chem..

[66] Ito S., Takeda A., Miyazaki T., Yokoyama Y., Saunders M., Cross R.J., Takagi H., Berthet P., Dragoe N. (2004). Kr extended X-ray absorption fine structure study of endohedral Kr@C_60_. J. Phys. Chem. B.

[67] Syamala M.S., Cross R.J., Saunders M. (2002). ^129^Xe NMR spectrum of xenon inside C_60_. J. Am. Chem. Soc..

[68] Bloodworth S., Whitby R.J. (2022). Synthesis of endohedral fullerenes by molecular surgery. Commun. Chem..

[69] Komatsu K., Murata M., Murata Y. (2005). Encapsulation of molecular hydrogen in fullerene C_60_ by organic synthesis. Science.

[70] Morinaka Y., Tanabe F., Murata M., Murata Y., Komatsu K. (2010). Rational synthesis, enrichment, and ^13^C NMR spectra of endohedral C_60_ and C_70_ encapsulating a helium atom. Chem. Commun..

[71] Kurotobi K., Murata Y. (2011). A single molecule of water encapsulated in fullerene C_60_. Science.

[72] Krachmalnicoff A., Bounds R., Mamone S., Alom S., Concistrè M., Meier B., Kouřil K., Light M.E., Johnson M.R., Rols S. (2016). The dipolar endofullerene HF@C_60_. Nat. Chem..

[73] Bloodworth S., Sitinova G., Alom S., Vidal S., Bacanu G.R., Elliott S.J., Light M.E., Herniman J.M., Langley G.J., Levitt M.H. (2019). First synthesis and characterization of CH_4_@C_60_. Angew. Chem. Int. Ed..

[74] Bloodworth S., Hoffman G., Walkey M.C., Bacanu G.R., Herniman J.M., Levitt M.H., Whitby R.J. (2020). Synthesis of Ar@C_60_ using molecular surgery. Chem. Commun..

[75] Hoffman G., Walkey M.C., Gräsvik J., Bacanu G.R., Alom S., Bloodworth S., Light M.E., Levitt M.H., Whitby R.J. (2021). A solid-state intramolecular Wittig reaction enables efficient synthesis of endofullerenes including Ne@C_60_, ^3^He@C_60_, and HD@C_60_. Angew. Chem. Int. Ed..

[76] Hoffman G., Bacanu G.R., Marsden E.S., Walkey M.C., Sabba M., Bloodworth S., Tizzard G.J., Levitt M.H., Whitby R.J. (2022). Synthesis and ^83^Kr NMR spectroscopy of Kr@C_60_. Chem. Commun..

[77] Dolgonos G.A., Peslherbe G.H. (2014). Encapsulation of diatomic molecules in fullerene C_60_: Implications for their main properties. Phys. Chem. Chem. Phys..

[78] Meier B., Mamone S., Concistre M., Alonso-Valdesueiro J., Krachmalnicoff A., Whitby R.J., Levitt M.H. (2015). Electrical detection of ortho–para conversion in fullerene-encapsulated water. Nat. Commun..

[79] Shugai A., Nagel U., Murata Y., Li Y., Mamone S., Krachmalnicoff A., Alom S., Whitby R.J., Levitt M.H., Rõõm T. (2021). Infrared spectroscopy of an endohedral water in fullerene. J. Chem. Phys..

[80] Ensing B., Costanzo F., Silvestrelli P.L. (2012). On the polarity of buckminsterfullerene with a water molecule inside. J. Phys. Chem. A.

[81] Aoyagi S., Hoshino N., Akutagawa T., Sado Y., Kitaura R., Shinohara H., Sugimoto K., Zhang R., Murata Y. (2014). A cubic dipole lattice of water molecules trapped inside carbon cages. Chem. Commun..

[82] Suzuki H., Nakano M., Hashikawa Y., Murata Y. (2019). Rotational Motion and Nuclear Spin Interconversion of H_2_O Encapsulated in C_60_ Appearing in the Low-Temperature Heat Capacity. J. Phys. Chem. Lett..

[83] Beduz C., Carravetta M., Chen J.Y.-C., Concistrè M., Denning M., Frunzi M., Horsewill A.J., Johannessen O.G., Lawler R., Lei X. (2012). Quantum rotation of ortho and para-water encapsulated in a fullerene cage. Proc. Natl. Acad. Sci. USA.

[84] Felker P.M., Vlček V., Hietanen I., FitzGerald S., Neuhauser D., Bačić Z. (2017). Explaining the symmetry breaking observed in the endofullerenes H_2_@C_60_, HF@C_60_, and H_2_O@C_60_. Phys. Chem. Chem. Phys..

[85] Ge M., Nagel U., Hüvonen D., Rõõm T., Mamone S., Levitt M.H., Carravetta M., Murata Y., Komatsu K., Chen J.Y.-C. (2011). Interaction potential and infrared absorption of endohedral H_2_ in C_60_. J. Chem. Phys..

[86] Saroj A., Ramanathan V., Mishra B.K., Panda A.N., Sathyamurthy N. (2022). Improved Estimates of Host-Guest Interaction Energies for Endohedral Fullerenes Containing Rare Gas Atoms, Small Molecules, and Cations. ChemPhysChem.

[87] Morinaka Y., Sato S., Wakamiya A., Nikawa H., Mizorogi N., Tanabe F., Murata M., Komatsu K., Furukawa K., Kato T. (2013). X-ray observation of a helium atom and placing a nitrogen atom inside He@C_60_ and He@C_70_. Nat. Commun..

[88] Aoyagi S., Nishibori E., Sawa H., Sugimoto K., Takata M., Miyata Y., Kitaure R., Shinohara H., Okada H., Sakai T. (2010). A layered ionic crystal of polar Li@C_60_ superatoms. Nat. Chem..

[89] Matsuo Y., Okada H., Ueno H. (2017). Endohedral Lithium-Containing Fullerenes Preparation, Derivatization, and Application.

[90] Okada H., Komuro T., Sakai T., Matsuo Y., Ono Y., Omote K., Yokoo K., Kawachi K., Kasama Y., Ono S. (2012). Preparation of Endohedral Fullerene Containing Lithium (Li@C_60_) and Isolation as Pure Hexafluorophosphate Salt ([Li^+^@C_60_][PF6-]). RSC Adv..

[91] Aoyagi S., Sado Y., Nishibori E., Sawa H., Okada H., Tobita H., Kasama Y., Kitaura R., Shinohara H. (2012). Rock-salt-type crystal of thermally contracted C_60_ with encapsulated lithium cation. Angew. Chem. Int. Ed..

[92] Aoyagi S., Tokumitu A., Sugimoto K., Okada H., Hoshino N., Akutagawa T. (2016). Tunneling motion and antiferroelectric ordering of lithium cations trapped inside carbon cages. J. Phys. Soc. Jpn..

[93] Bai H., Gao H., Feng W., Zhao Y., Wu Y. (2019). Interaction in Li@Fullerenes and Li^+^@Fullerenes: First Principle Insights to Li-Based Endohedral Fullerenes. Nanomaterials.

[94] Noguchi Y., Sugino O., Okada H., Matsuo Y. (2013). First-principles investigation on structural and optical properties of M^+^@C_60_ (Where M = H, Li, Na, and K). J. Phys. Chem. C.

[95] Koner A., Kumar C., Sathyamurthy N. (2018). Heat capacity of endohedral fullerenes Rg@C_60_ (Rg = He, Ne, Ar and Kr). Mol. Phys..

[96] Pyykkö P., Wang C., Straka M., Vaara J. (2007). A London-type formula for the dispersion interactions of endohedral A@B systems. Phys. Chem. Chem. Phys..

[97] Proft F.D., Alsenoy C.V., Geerlings P. (1996). Ab Initio Study of the Endohedral Complexes of C_60_, Si_60_, and Ge_60_ with Monoatomic Ions: Influence of Electrostatic Effects and Hardness. J. Phys. Chem..

[98] Shameema O., Ramachandran C.N., Sathyamurthy N. (2006). Blue Shift in X-H Stretching Frequency of Molecules Due to Confinement. J. Phys. Chem. A.

[99] Wang C., Straka M., Pyykkö P. (2010). Formulations of the closed-shell interactions in endohedral systems. Phys. Chem. Chem. Phys..

[100] Jaworski A., Hedin N. (2021). Local energy decomposition analysis and molecular properties of encapsulated methane in fullerene (CH_4_@C_60_). Phys. Chem. Chem. Phys..

[101] Darzynkiewicz R.B., Scuseria G.E. (1997). Noble Gas Endohedral Complexes of C_60_ Buckminsterfullerene. J. Phys. Chem. A.

[102] Oliveira O.V.d., da Silva Gonçalves A. (2014). Quantum Chemical Studies of Endofullerenes (M@C_60_) Where M = H_2_O, Li^+^, Na^+^, K^+^, Be^2+^, Mg^2+^, and Ca^2+^. Comput. Chem..

[103] Hu Y.H., Ruckenstein E. (2005). Endohedral Chemistry of C_60_-Based Fullerene Cages. J. Am. Chem. Soc..

[104] Slanina Z., Pulay P., Nagase S. (2006). H_2_, Ne, and N_2_ Energies of Encapsulation into C_60_ Evaluated with the MPWB1K Functional. J. Chem. Theory Comput..

[105] Kalugina Y.N., Roy P.-N. (2017). Potential energy and dipole moment surfaces for HF@C_60_: Prediction of spectral and electric response properties. J. Chem. Phys..

[106] Kruse H., Grimme S. (2009). Accurate Quantum Chemical Description of Non-Covalent Interactions in Hydrogen Filled Endohedral Fullerene Complexes. J. Phys. Chem. C.

[107] Gao H., Sun Y., Zhang J., Wang Q., Wu Y., Bai H. (2021). Understanding endohedral behaviors of ten-electron atomic and cluster system inside C_60_ from first-principles. Phys. Low-Dimens. Syst. Nanostructures.

[108] Remya P.R., Mishra B.K., Ramachandran C.N., Sathyamurthy N. (2019). Effect of confinement on structure, energy and vibrational spectra of (HF)_n_, n=1–4. Chem. Phys. Lett..

[109] See for example: Delaney P., Greer J.C. (2004). C_60_ as a Faraday cage. Appl. Phys. Lett..

[110] Burger S., Lipparini F., Gauss J., Stopkowic S. (2021). NMR chemical shift computations at second-order Møller–Plesset perturbation theory using gauge-including atomic orbitals and Cholesky-decomposed two-electron integrals. J. Chem. Phys..

[111] Cioslowski J., Rao N., Pernal K., Moncrieff D. (2003). Endohedral motions inside capped single-walled carbon nanotubes. J. Chem. Phys..

